# Review on Phase Synchronization Methods for Spaceborne Multistatic Synthetic Aperture Radar

**DOI:** 10.3390/s24103122

**Published:** 2024-05-14

**Authors:** Qiang Lin, Shiqiang Li, Weidong Yu

**Affiliations:** 1 Department of Space Microwave Remote Sensing System, Aerospace Information Research Institute, Chinese Academy of Sciences, Beijing 100190, China; linqiang21@mails.ucas.ac.cn (Q.L.); lishq@mail.ie.ac.cn (S.L.); 2 School of Electronic, Electrical and Communication Engineering, University of Chinese Academy of Sciences, Beijing 100049, China

**Keywords:** spaceborne multistatic SAR, phase synchronization, frequency offset

## Abstract

Multistatic synthetic aperture radar (SAR) is a special mode of SAR system. The radar transmitter and receiver are located on different satellites, which brings many advantages, such as flexible baseline configuration, diverse receiving modes, and more detailed ground object classification information. The multistatic SAR has been widely used in interferometry, moving target detection, three-dimensional imaging, and other fields. The frequency offset between different oscillators will cause a modulation phase error in the signal. Therefore, phase synchronization is one of the most critical problems to be addressed in distributed SAR systems. This article reviews phase synchronization techniques, which are mainly divided into two methods: synchronization by direct microwave link and synchronization by a data-based estimation algorithm. Furthermore, the future development of synchronization technology is anticipated.

## 1. Introduction

**Figure 1:**
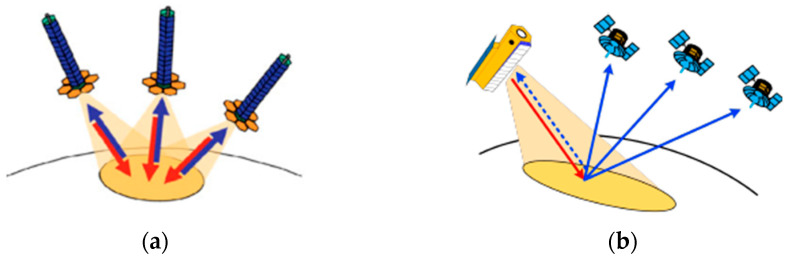
The type of multistatic SAR system. (**a**) Fully active SAR system; (**b**) Semi-active SAR system.

SAR can provide high-resolution images in all-weather and all-day conditions; it provides high-resolution data for global remote sensing, terrain surveying, deformation monitoring, and other applications [1]. The characteristic of spaceborne multistatic SAR systems is the installation of transmitters and receivers on separate platforms. The multistatic SAR systems are classified into two types based on whether each platform in the system has the capability to transmit radar signals and receive echoes: fully active systems and semi-active systems. In fully active systems, as shown in Figure 1a, each satellite in the multistatic system has the capability to both transmit radar signals and receive echoes. Therefore, each satellite can autonomously acquire monostatic images or solely receive echo signals to obtain bistatic images. This type offers advantages such as flexible baseline configuration, multi-angle observation, and diverse formation configurations. In the semi-active systmes, as shown in Figure 1b, only one or some of the satellites in the multistatic system have transmitting capabilities, while the rest of the satellites only have receiving capabilities. Consequently, compared to fully active systems, their flexibility is reduced. However, the semi-active systems also have their unique advantages: satellites that solely receive signals can be implemented using smaller satellites, leading to a significant cost reduction. The receivers do not need to transimit a signal, so the concealment ability is strong. The multistatic SAR, as a highly versatile remote sensing technology, will play a significant role in military reconnaissance, environmental monitoring, dynamic Earth measurement, and ocean surveillance, among other fields. Since the 1990s, multistatic SAR systems have gained increasing attention from researchers and have received widespread research [2,3,4,5].

**Figure 2:**
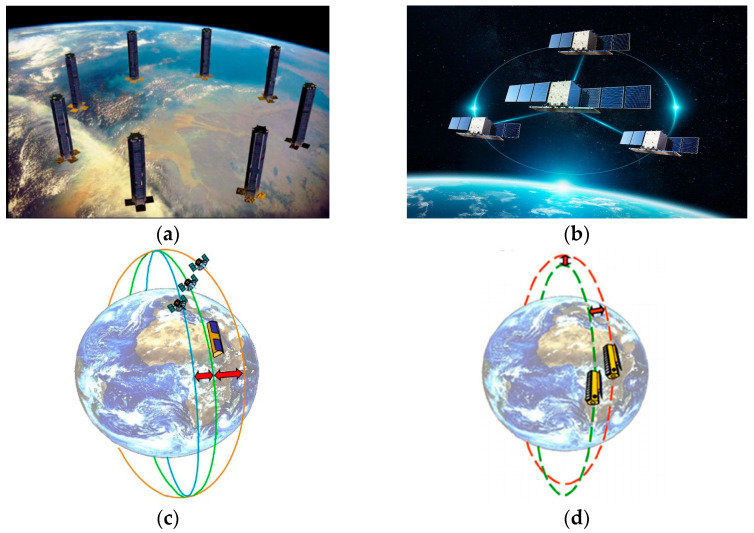
Schematic diagram of formation configuration: (**a**) TechSat-21 formation configuration [7]; (**b**) cartwheel formation configuration; (**c**) pendulum formation configuration [5]; (**d**) dual-helix formation configuration [3].

In the mid-1990s, the concept of “virtual satellite” was proposed, i.e., multiple small satellites with relatively single functions work together to achieve the functions of a traditional large satellite [6]. The U.S. Air Force Research Laboratory‘s Techsat-21 program in 1998 [7] officially proposed the concept of spaceborne multistatic SAR. The configuration diagram of the Techsat-21 is shown in Figure 2a. The purpose is mainly battlefield surveillance, slow vehicle tracking on the ground, and ground motion target detection. At the beginning of the 21st century, the French National Space Research Center proposed the interferometric cartwheel program [8,9]. The formation configuration is shown in Figure 2b. The plan is to form a cartwheel formation with three small satellites responsible for receiving signals and a parallel-track formation with a main satellite. Then, the signals received by the three small satellites are imaged, and three-dimensional terrain information is obtained through three interferograms. The German Aerospace Center (DLR) proposed the interferometric pendulum plan based on the interferometric wheel plan [5]. Different from the cartwheel plan, the relative motion trajectories of the three small satellites are parallel to the ground plane. The baseline along the heading is fixed, and the distance baseline can be flexibly adjusted in a pendulum plan, making it more suitable for marine applications. Its configuration is shown in Figure 2c.

The TanDEM-X satellite, launched by DLR in 2010 [10], formed a bistatic SAR system with the TerraSAR-X satellite. Two satellites fly in formation in the vertical heading plane with a “spiral” trajectory, and their configuration is shown in Figure 2d. TanDEM-X has been in operation since the launch of satellite [3], which opened a new area for spaceborne multistatic SAR. The TianHui-2 satellite was launched in April 2019 [11]. Both satellites use GNSS to tame crystal oscillators to generate high-stable reference frequencies. At the same time, high-precision rubidium atomic clocks are introduced as frequency references in inter-satellite measurement equipment to further improve frequency accuracy. The LuTan-1 satellite system, consisting of two satellites with a full-polarimetric L-band SAR satellite, is the first Earth observation bistatic SAR system in the world whose primary mission is surface deformation monitoring. Both satellites were launched in early 2022 [12,13,14].

In addition, there are also many distributed SAR system tasks. In 2009, followed by TanDEM-X, DLR’s Moreira et al. designed the TanDEM-L task. TanDEM-L uses specially designed acquisition modes and advanced digital beam-forming technology to perform global observations of dynamic processes on the Earth’s surface with unprecedented quality and resolution [15,16]. In 2017, Krieger et al. of DLR proposed a “MirrorSAR” concept for high-resolution wide-swath SAR imaging. The target scene is illuminated by one or more transmitter satellites. The scattered radar waves are then spatially sampled by multiple receivers that route their recorded signals to the transmitter. The transmitter satellite, in turn, coherently demodulates and combines the multiple forwarded signals, before the relevant information is transferred to the ground [17,18]. In 2017, DLR and ESA jointly proposed the SAOCOM-CS task [19,20]. This task mainly combines the existing SAR satellite with one or more other miniature SAR satellites to build a distributed SAR system along the track. Among them, SAOCOM stated that the L-band of Argentine SAOCOM-1B and CS indicate that the SAR satellite with only passive receiving capabilities is planned to launch. In 2019, the SESAME mission and the Harmony concept were presented jointly by Paco et al. from the Netherlands and scholars from DLR. SESAME is a new C-band tomography system that combines Sentinel-1 as a launching source with two additional close-range SAR satellites launched in formation [21]. Harmony is a pre-launched Sentinel-1D with additional satellites to form a single ATI or XTI distributed SAR system [22]. These successively proposed spaceborne distributed SAR missions will make great contributions to Earth observation in the future.

In addition to the systems with the spaceborne–spaceborne configuration described previously, spaceborne multistatic SAR systems also include spaceborne–airborne configurations, spaceborne-stationary configurations, and non-cooperative multistatic SAR systems. The spaceborne–airborne/stationary configuration is a BiSAR system with a spaceborne transmitter and an airborne/ground-based receiver. A series of spaceborne–airborne experiments were conducted with TerraSAR-X as the transmitter, and F-sar and PAMIR as the receivers. Digital elevation model reconstruction in multichannel spaceborne–stationary SAR interferometry and the first bistatic demonstration of digital beamforming in elevation with TerraSAR-X as an illuminator were conducted in [23,24], respectively. Non-cooperative multistatic SAR system, using the global navigation satellite system (GNSS) or other public signals as the transmitting source Experiments of bistatic SAR that use GNSS as transmitters have been conducted in [25,26,27,28].

**Figure 3:**
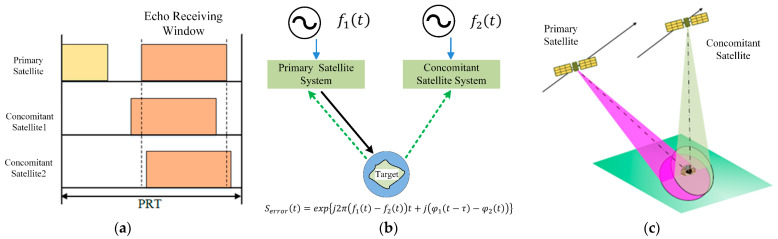
Synchronization in multistatic systems: (**a**) time synchronization; (**b**) phase synchronization; (**c**) beam synchronization.

Not only does a spaceborne multistatic SAR system bring many advantages, it also puts forward high requirements for its system design and brings many challenges. One of the challenges in a multistatic SAR system is synchronization [3,4,12,14,29,30,31,32,33,34,35,36,37], as shown in Figure 3. The time synchronization problem refers to the fact that the receiving and transmitting radars use different crystal oscillators to generate pulse signals, which causes inconsistency in the trigger pulse, resulting in inconsistency between the time of the start of the radar signal and the starting time of the radar echo-receiving windows, so that the echo signal cannot be recorded correctly. Furthermore, this deviation accumulates with time, which will significantly affect the quality of images. Time synchronization can be achieved by using a highly stable frequency source and a GPS pulse-per-second (PPS) tamed crystal oscillator [38].

The beam synchronization problem refers to the fact that the receiving and transmitting antennas cannot simultaneously illuminate the same spot on the ground, resulting in a decrease in the bistatic antenna pattern gain, and the receiving antennas cannot obtain all the target scattered signals, resulting in a loss of effective swath width. To ensure system performance, beam synchronization can be achieved by precisely controlling the beam pointing of the antenna.

The difference in crystal frequency that leads to the inconsistency between the modulation frequency of the transmit signal and the demodulation frequency of the receiving signal causes a residual modulation of the recorded SAR raw data, which is referred to as phase synchronization. Therefore, phase synchronization must be achieved to ensure that the receiver and transmitter are coherent for an extremely long time. Actually, in practice, phase synchronization is the most challenging of these synchronization problems. Much effort has been dedicated in recent years to the investigation of phase synchronization strategies for distributed SAR. Possible solutions range from the use of dedicated calibration devices to the use of dedicated processing techniques. The phase synchronization is realized by a microwave direct link [32,39]. For example, in the TanDEM-X mission, synchronization with this level of accuracy was achieved by exchanging radar pulses between the satellites through a direct microwave link. Another possible method could be the estimation of the synchronization phase based on the evaluation of the received data, such as autofocus, which can be used in the absence of dedicated hardware solutions, as in the case of the SAOCOM-CS mission [20]; In addition, to acquire frequency deviations, some algorithms based on the data estimation of the navigation receiver are proposed to achieve phase synchronization [40]. This article reviews the phase synchronization method and its applications in distributed SAR systems.

## 2. Phase Synchronization in Multistatic SAR

Multistatic SAR systems are characterized by a separate transmitter and receiver mounted on different platforms. Since the modulation frequency of the transmitted signal is inconsistent with the demodulation frequency of the received signal, a phase error of the demodulated echo along the azimuth time direction is caused.

In the spaceborne multistatic SAR system, the phase error of the oscillator can be expressed as

(1) $$\phi \left(t\right)=2\pi f_{o}\left(t\right)t+\phi_{st}\left(t\right)+\phi_{0}$$

where $f_{o}\left(t\right)$ is the frequency offset resulting in a linear phase (ideally a fixed value different from the nominal frequency), $\phi_{0}$ is the arbitrarily fixed phase offset of each oscillator, and $\phi_{st}$ is the phase noise, which can be modeled by a second-order stationary stochastic process.

This stationary stochastic process is usually described by power spectrum density (PSD). The power spectrum density of the oscillator phase noise can be represented by a composite power model [36,41,42],

(2) $$S_{\phi ,osc}^{TS}\left(f\right)=\sum_{\alpha =0}^{4}h_{\alpha }f^{-\alpha }$$

where, coefficients $h_{4}$–$h_{0}$ describe contributions from (1) random walk frequency noise, (2) flicker frequency noise, (3) white frequency noise, (4) flicker phase noise, and (5) white phase noise, respectively.

**Figure 4:**
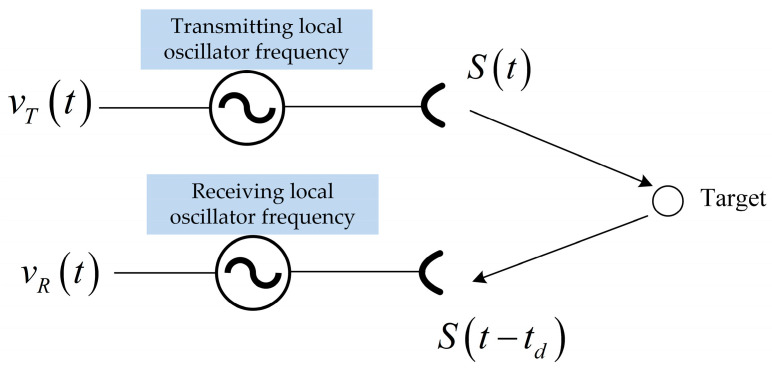
The multistatic SAR system phase error generation diagram.

Figure 4 shows a diagram of the effect of bistatic SAR oscillator noise on radar echo. The radar pulse is modulated by the transmitting local oscillator signal $v_{T}\left(t\right)$ and transmitted, reflected by the target, received by the receiver, and demodulated by the receiving local oscillator signal $v_{R}\left(t\right)$. Without considering the amplitude variation of the oscillator, frequency drift, and the effect of environmental changes, the output of the receiver and transmitter frequency sources of the bistatic SAR can be expressed as

(3) $$v_{T}\left(t\right)=cos\left(2\pi f_{T}t+\phi_{st\_T}\left(t\right)\right)$$

(4) $$v_{R}\left(t\right)=cos\left(2\pi f_{R}t+\phi_{st\_R}\left(t\right)\right)$$

where $f_{T}$, $\phi_{st\_T}\left(t\right)$ is the center frequency and phase noise of the transmitter carrier, respectively; $f_{R},\;\phi_{st\_R}$ is the center frequency and phase noise of the received carrier, respectively. They are obtained by multiplying the oscillator frequency by m, where $m=f_{0}/f_{osc}$ is the frequency up-conversion factor and $f_{0}$ is the radar carrier frequency.

The delay of the echo from the transmitter to the receiver through the target reflection is τ, the echo at time t is the transmitted signal at time t−τ. Therefore, the phase error due to the oscillator can be represented as [36,43]

(5) $$\Phi \left(t\right)=-2\pi f_{T}\tau +2\pi \left(f_{T}-f_{R}\right)t+\left[\phi_{st\_T}\left(t-\tau \right)-\phi_{st\_R}\left(t\right)\right]$$

The first term in (3) denotes the echo delay information used for SAR imaging. The second term is the linear error term caused by the initial frequency deviation. It has no effect on image focusing and may cause range and azimuth under sampling of bistatic SAR. The third term is the phase error caused by phase noise. The latter two terms are echo phase errors caused by oscillator phase noise, which are the focus of phase synchronization research.

The synchronization phase error caused by the phase noise between the two oscillators can be expressed as

(6) $$\Delta \Phi_{syn\_error}\left(t\right)=\phi_{st\_T}\left(t-\tau \right)-\phi_{st\_R}\left(t\right)$$

Supposing that $\phi_{s{t\_}_{T}}(t)$, $\phi_{s{\_t}_{R}}(t)$ are two independent stationary stochastic processes that have the same power spectral density, the psd of the phase noise of the bistatic SAR echo can be expressed as

(7) $$S_{\phi \_B}\left(t\right)=2m^{2}S_{\phi ,osc}\left(f\right)$$

Equation (7) shows that the bistatic SAR echo phase error is the direct addition of the transmitted and received frequency phase noise. In the monostatic system, the same oscillator is used for receiving and transmitting, therefore, the PSD of phase noise of the monostatic SAR echo can be expressed as

(8) $$S_{\phi \_M}\left(t\right)=m^{2}\left(2sin\left(\pi f\tau \right)\right)S_{\phi ,osc}\left(f\right)$$

where τ is the echo delay. Equation (8) shows that the oscillator phase noise is almost cancelled out, and the amount of cancellation depends on the signal propagation time τ, which is negligible in practice.

**Figure 5:**
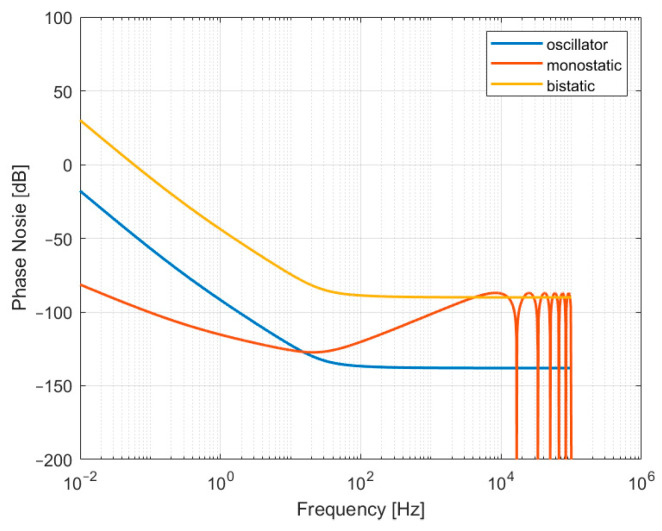
Phase error power spectrum of monostatic and bistatic SAR echoes generated by oscillator phase noise. $S_{\phi ,osc}\left(f\right)$ uses $\left\{h_{4}=-95\;dB,\;h_{3}=-90\;dB,\;h_{2}=-200\;dB,\;h_{1}=-130\;dB,\;h_{0}=-155\;dB\right\}$.

Figure 5 shows the PSD of the phase error of the monostatic and bistatic SAR echoes generated by the phase noise of the oscillator. In the monostatic system, the low frequency component of the oscillator phase noise is suppressed, and the high frequency component is enhanced, but the magnitude is in a small order in general. In the bistatic system, the phase error can be considered the amplification of the oscillator phase noise, especially the low frequency. The magnitude is already relatively large, and the amplification again may have a great impact on bistatic SAR imaging. In multistatic SAR signal processing, the oscillator phase noise may not only defocus the SAR image in the azimuth but also introduce significant positioning and phase errors along the scene extension. and the phase error will eventually be transferred to the interference phase, resulting in the loss of interferometric accuracy [30,36]. In addition, the quality of phase synchronization is evaluated by the residual phase error in a spaceborne multistatic SAR system.

**Figure 6:**
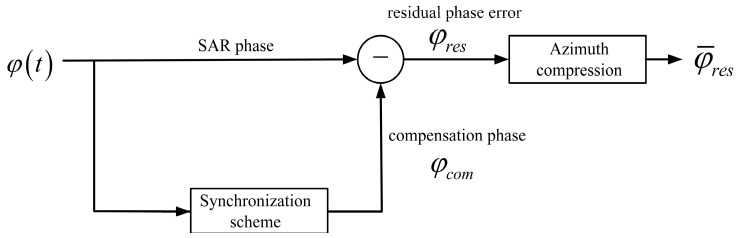
Phase synchronization and SAR processing flow block diagram.

In addition, in a spaceborne multistatic SAR system, the quality of phase synchronization is evaluated by the residual phase error. Before SAR focusing, the general flow block diagram of the phase synchronization scheme is shown in Figure 6. The residual phase error will be further reduced after imaging processing. Because, during azimuth pulse compression, the residual phase error is averaged over a synthetic aperture time $T_{a}$, equivalent to passing through a low-pass filter. Finally, the phase error propagated to the interferometric processing can be represented as ${\bar{\varphi }}_{res}$. In interference processing, the interferometric phase error [3] not only includes errors arising from baseline decorrelation, volume decorrelation, temporal decorrelation, etc., but also encompasses residual phase error. However, the residual phase error is relatively small compared to other interferometric phase errors.

It should be noted that in addition to the phase error caused by the oscillator, system noise, ionospheric effects, multipath effects, and so on can also cause phase errors. In this paper, we focus on the phase error introduced by the oscillator error, and other factors are beyond the scope of this paper.

## 3. Phase Synchronization Methods

### 3.1. Synchronization by Direct Microwave Link

A characteristic of data acquisition in multistatic SAR is that the radar pulse is modulated and demodulated by different independent oscillators. Therefore, the fixed frequency offset and phase noise of the oscillators will cause phase errors in azimuth signals.

**Figure 7:**
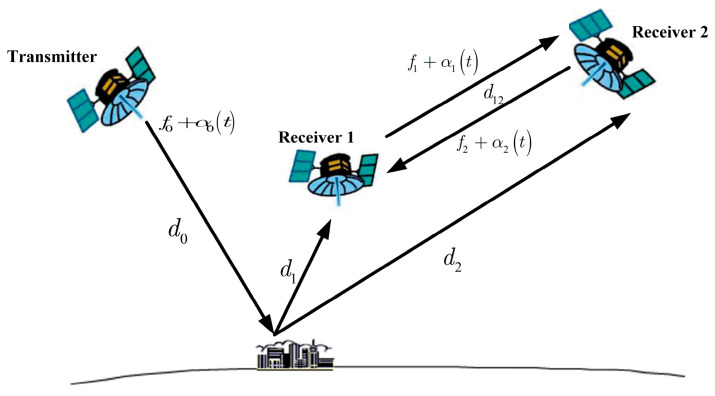
Synchronization scheme proposed in [39].

The phase error caused by the oscillator can be compensated by a dedicated synchronization link. Synchronization by direct microwave link can be divided into continuous duplex synchronization, pulse duplex synchronization, and pulse alternate synchronization. The continuous duplex synchronization method was first proposed in [39]. As shown in Figure 7, a radar signal is transmitted by a transmitter, and the echo is received by two receivers with a certain baseline. At the same time, the two receivers transmit oscillator signals to each other. The phase noise caused by oscillator errors can be compensated by the exchanged oscillator phase information. The phase error caused by the relative motion of the receiving satellites can also be compensated.

Synchronization methods by direct microwave link have been further investigated; pulse duplex synchronization and pulse alternate synchronization were proposed in [31]. Pulse duplex synchronization is similar to continuous duplex synchronization, which replaces local oscillator signals with synchronization pulses. Thus, their system hardware must be able to transmit and receive synchronization signals at the same time, and the signals must be sufficiently decoupled. In the pulse alternate synchronization method, synchronization pulses are transmitted alternately. Compared with duplex synchronization methods, the synchronization signal is decoupled, and a single carrier frequency can possibly be used. Pulse alternate synchronization has been applied to the multistatic SAR system. The principle of phase synchronization by direct microwave link is to extract the compensation phase from the exchanged synchronization signals. Therefore, this method can be represented by a general mathematical model. Supposing that satellite 1 transmits the synchronization signal, the signal received by satellite 2 can be written as

(9) $$\begin{array}{cc} & \Phi_{1}\left(t\right)=\phi_{T}\left(t-\tau_{syn}\right)-\phi_{R}\left(t\right)\\ = & -2\pi f_{T}\tau_{syn}+2\pi \left(f_{T}-f_{R}\right)t+\left[\phi_{st\_T}\left(t-\tau \right)-\phi_{st\_R}\left(t\right)\right] \end{array}$$

where $\tau_{syn}$ is the synchronization signal delay. The transmitter of Satellite 2 also transmits the synchronization signal to the receiver of Satellite 1, and the received phase of Satellite 1 can be written as

(10) $$\begin{array}{cc} & \Phi_{2}\left(t\right)=\phi_{R}\left(t-\tau_{syn}\right)-\phi_{T}\left(t\right)\\ = & -2\pi f_{R}\tau_{syn}+2\pi \left(f_{R}-f_{T}\right)t+\left[\phi_{st\_R}\left(t-\tau \right)-\phi_{st\_T}\left(t\right)\right] \end{array}$$

Therefore, the compensation phase can be obtained by

(11) $$\begin{array}{c} {∆\Phi }_{com}\left(t\right)=\frac{1}{2}\left(\Phi_{1}\left(t\right)-\Phi_{2}\left(t\right)\right)\\ \approx 2\pi \left(f_{T}-f_{R}\right)t+\left[\phi_{st\_T}\left(t\right)-\phi_{st\_R}\left(t\right)\right] \end{array}$$

Approximations $\phi_{st\_R}\left(t-\tau \right)\approx \phi_{st\_R}\left(t\right)$ and $\phi_{st\_T}\left(t-\tau \right)\approx \phi_{st\_T}\left(t\right)$ are used in (9) and (10), respectively, because $\tau_{syn}$ is generally very small in the system.

The difference between compensated phase and phase synchronization error is the echo delay. The echo delay is small and can be ignored in the analysis. Thus, the compensated phase compensates with high precision for the phase error introduced by the oscillator. In addition, the phase difference value is used as the compensated phase, so long as the errors caused by the antenna, Tx/Rx system phase changes, and link path do not change during the transmission of synchronization signals, then these errors are also offset.

**Figure 8:**
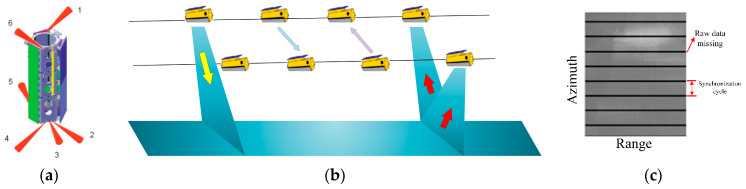
(**a**) the configuration and orientation of the synchrohorn on TDX [46]; 1-6 represents 6 synchronization antennas; (**b**) pulse exchange synchronization of TanDEM-X satellites [5]; (**c**) some echo data were missing.

Currently, multistatic SAR systems on orbit all use the pulse alternate synchronization method to achieve phase synchronization. Pulse alternate synchronization methods are divided into interrupted synchronization methods and noninterrupted synchronization methods. In the DLR’s TanDEM-X/TerraSAR-X system, the interrupted pulsed alternate synchronization method is used. Both satellites are equipped with six circularly polarized X-band horn antennas, arranged as shown in Figure 8a, to obtain omnidirectional coverage and ensure phase synchronization of all possible satellite relative positions [3,5,44,45,46]. However, in order to transmit synchronization signals, the TanDEM-X system needs to interrupt the SAR imaging process synchronously, as shown in Figure 8b, resulting in periodic loss of original data [3]. Thus, it is necessary to reconstruct the missing original data [47,48]. To ensure imaging quality, excessive missing data should be avoided, which could result in a lower synchronization frequency. The errors affecting the compensation phase quality of interrupted pulsed alternate synchronization methods can be primarily classified into two categories: one is interpolation and aliasing errors caused by low synchronization frequency, and the other is the SNR of the synchronization link. For the typical DEM data acquisition modes with baselines less than 1 km, the SNR will be on the order of 30 to 40 dB, and phase errors lower than 1 degree can be achieved for synchronization frequencies higher than 5 Hz [3].

**Figure 9:**
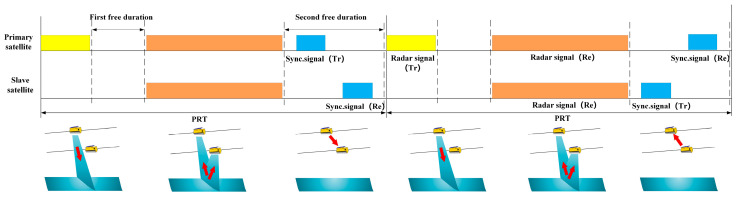
Timing diagrams of noninterrupted synchronization pulse exchange in the LuTan-1 mission [50].

To solve the problems in the TanDEM-X synchronization method, the noninterrupted synchronization method is proposed [49,50,51,52]. The timing diagram of the synchronization method is shown in Figure 9. There are two free times within a pulse repetition interval (PRI). The first is the free time between the end of the radar transmit signal and the start of the radar echo receiving window, Correspondingly, the second is the time between the end of the radar echo receiver window and the start of the next PRI. In the LuTan-1 mission, phase synchronization no longer needs to interrupt the SAR operation, and the synchronization signal is exchanged in the free time between the PRI, which avoids the loss of SAR data. At the same time, the synchronization frequency can reach up to PRF/2 (about 1400 Hz in LuTan-1), far exceeding the synchronization frequency of interrupt-driven synchronization methods (about 5 Hz in TerraSAR-X/TanDEM-X). This lays the foundation for achieving high-precision synchronization phases. The error affecting the compensation phase quality of the noninterrupted pulsed alternate synchronization method is the SNR of the synchronization link. When the SNR is greater than 60 dB, the standard deviation of residual phase error is less than 0.1 degree [50]. Although noninterrupted phase synchronization methods avoid interrupting the SAR imaging process, the flexibility in the design of echo reception time $T_{ecoh}$ is limited. If the transmission time of the synchronization signal exceeds the echo reception time, the bandwidth will inevitably be affected. Therefore, synchronization by direct microwave link needs further research.

It is worth noting that while using dedicated hardware links to implement phase synchronization, new phase errors are also introduced, such as hardware system phase drift, Doppler phase error, antenna pattern errors, etc. References [31,50] derive and analyze the errors existing in the synchronization link. Reference [53] first demonstrates the impact of multipath effects on the LT-1 phase synchronization scheme, indicating that phase synchronization can still be achieved under multipath effects. This is because the time required to implement a pulse exchange in the phase synchronization scheme is extremely short, as is the reversibility of electromagnetic propagation. The impact of multipath effects on bistatic synchronization signals may be similar. In the process of calculating the compensation phase, subtraction processing is required for the extracted bistatic synchronization phase, which offsets the effect of multipath effects. For the ionospheric effects, generally, satellites operate above 500 km, where the ionosphere has little impact within this range. Additionally, in multistatic SAR systems, the distances between satellites are close, and the ionospheric density is relatively uniform among them. Hence, it can be assumed that the phase error caused by the ionosphere on the synchronization signal of each satellite is basically the same. The phase difference (the compensation phase is actually the difference of the difference) further reduces ionospheric errors. Therefore, the impact of the ionosphere can be considered negligible.

In addition, when bistatic SAR works in alternating bistatic/ping-pong mode, phase synchronization can be realized directly through an echo signal without a direct microwave link. An echo domain phase synchronization method in bistatic/ping-pong alternate mode is proposed in [3]. Based on these studies, the sub-aperture processing method and the echo domain processing method are further studied in [54,55].

### 3.2. Synchronization by Data-Based Estimation Algorithm

Synchronization by using the direct microwave link can achieve high-accuracy phase synchronization in multistatic systems. However, it is achieved at the expense of designing additional hardware within the SAR system. Besides the need for additional hardware, the integration of synchronization by direct microwave link may have problems due to the different development times of different constellation elements, as is the case with companion SAR missions. Furthermore, the synchronization accuracy is also affected by the SNR of the synchronization pulse in the formation of the big baseline, which may also present challenges for this direct link synchronization method. In response to the above situations, autonomous estimation synchronization methods by autofocus and multisquint processing are proposed in [56]. Both methods achieve phase synchronization based on the estimation of the received data.

SAR autofocus algorithms are one of the key technologies to improve SAR imaging quality. It can estimate residual phase errors in the SAR image caused by factors such as the movement of the radar platform and changes in atmospheric conditions. In bistatic SAR images, phase synchronization errors caused by oscillators can also be considered a type of residual phase error. Therefore, autofocus algorithms can be used to estimate the positioning and phase distortions in bistatic SAR images. These distortions can then be used to estimate clock phase errors, which can achieve phase synchronization of bistatic data. In recent decades, many focusing algorithms have been proposed, such as Map Drift (MD) [57], Phase Difference (PD) [58,59] and phase gradient automatic focusing (PGA) [60]. The Map Drift (MD) algorithm divides an aperture into two nonoverlapping sub-apertures and can estimate the quadratic phase error. The improved MD algorithm can also estimate the phase error of any order, with the disadvantage of being computationally intensive. The PD algorithm solves this problem by splitting the synthetic aperture into multiple sub-apertures of the same length. However, the MD and PD algorithms have the common disadvantage that the estimated phase error depends on a pre-established phase error function model. If the pre-established mathematical model does not match the real model, the estimation results will have a large error.

The PGA (Phase Gradient Autofocus) algorithm no longer relies on hypothetical mathematical models but on the detection and analysis of phase errors for opportune point targets [60]. The PGA technique estimates the phase error at each azimuth position by minimizing the phase gradient in the image domain. The quality of the error estimate depends on the number of available targets in the image and the signal-to-clutter ratio (SCR) of the targets. The standard deviation of clock phase error estimation using PGA in a bistatic system can be expressed as [61]

(12) $$ls_{\phi }\approx \frac{1}{I{\left[n_{a}\right]}^{2}}\sum_{i=0}^{I\left[n_{a}\right]-1}\left(\frac{k_{1}}{\sqrt{{SCR}_{i}}}+\frac{k_{2}}{\sqrt{{SCR}_{i}}}\right)$$

where $n_{a}$ is the discrete slow time variable, $I\left[n_{a}\right]$ is the number of targets of opportunity. SCR is the signal-to-noise ratio for each target, $k_{1}$ and $k_{2}$ are constants with values 0.674 and 0.344, respectively. In the airborne bistatic experiment [61], the PGA technique was used to obtain a well-focused bistatic image.

Autofocus algorithms for residual estimation and compensation require a sufficient number of targets with a high SCR, so they are limited in practical applications. Multisquint processing is suitable for most situations and is more sensitive to residuals. The multisquint processing approach is suitable for most situations and is more sensitive to residuals. The multisquint processing method is used to obtain the desired phase correction by processing the same image pair with different squint angles and combining the information from different interferograms. The estimation of phase azimuth undulations by the multisquint processing method can be expressed as [62]

(13) $$\phi_{undul}\left(x,r\right)=\int \frac{\phi_{diff}\left(x,r\right)}{\Delta x\left(r\right)}dx+C$$

where $\phi_{undul}\left(x,r\right)$ is the phase of the differential interferogram, $\Delta x\left(r\right)$ is the distance between both acquisition geometries, and C is the unknown phase constant. Methods for estimating the residual phase error are described in detail in [63,64,65]. Then, an improved multisquint processing method is presented in [66], where the algorithm provides a robust estimation of the derivative of the baseline error over the whole image, even if some parts of the scene are fully de-correlated. A method that combines an inverse projection algorithm and a multisquint technique to estimate the residual phase error is given in [67]. A combination of the multisquint processing method and other processing methods to reduce errors caused by image artefacts is given in [68,69]. A method is provided in [70] to estimate and compensate for ionospheric perturbations and clock drift perturbations by using the multisquint processing method.

In addition, a method for the estimation of synchronization phase errors in the multistatic SAR with azimuth multi-channel was proposed in [71]. The algorithm shows stronger robustness to processing errors when processing unfocused SAR data. Autonomous synchronization has been used to evaluate data obtained in cross-platform bistatic interferometric experiments such as the DLR-ONERA two-base experiment [72] and the bistatic interferometric experiments of TanDEM-X [73].

### 3.3. Other Phase Synchronization Methods

#### 3.3.1. The Evaluation of Raw Data of the Navigation Receivers

**Figure 10:**
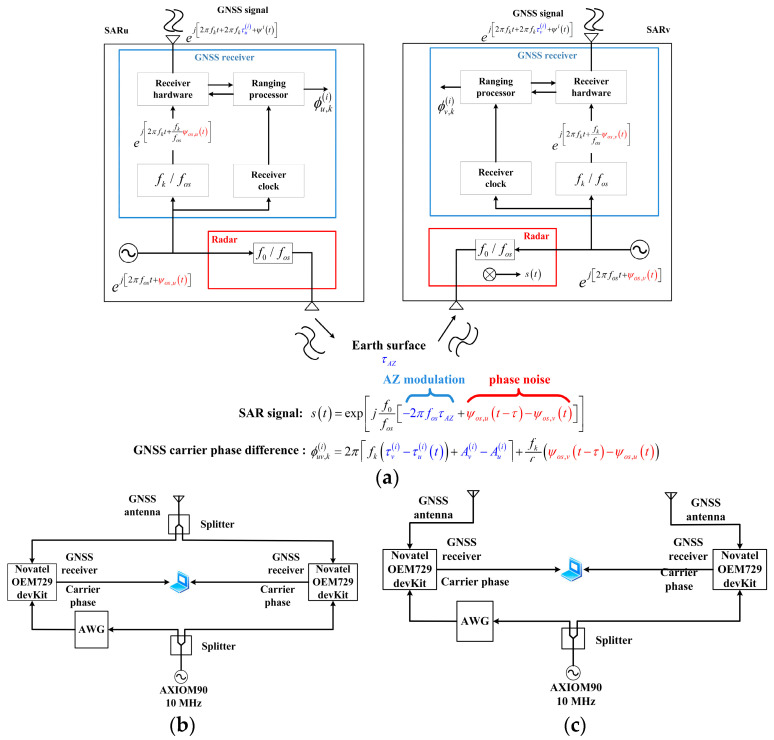
(**a**) Phase synchronization based on GNSS data [74]; (**b**) zero-base line phase and frequency synchronization experiment [40]; (**c**) short baseline phase and frequency synchronization experiments [40].

Spaceborne multistatic SAR systems play an increasingly important role in HRWS imaging and high precision interference, and phase synchronization has been studied more extensively. New phase synchronization methods are constantly being proposed. A synchronization method based on precise orbit determination (POD) and GNSS raw data is proposed in [74]. As shown in Figure 10a, this approach requires that the GNSS receiver and SAR payload share the same oscillator. The pseudo-distance between the primary satellites (u, v, and GNSS) is measured from the carrier phases received by the GNSS receiver. The true distance between the primary satellite, u, v, and the GNSS satellite is provided by POD. Then, the frequency offset and phase drift of the satellite oscillator are estimated by the double difference between the difference of pseudo distance between satellites u, v, and GNSS satellites and the difference of true distance between satellites u, v, and GNSS satellites. The estimate of the phase synchronization error obtained by using N navigation satellites and $n_{\lambda }^{\left(i\right)}$ GNSS frequencies can be expressed as:

(14) $$\psi_{uv}=\frac{2\pi }{\lambda }\sum_{i=1}^{N}\sum_{k=1}^{n_{\lambda }^{\left(i\right)}}\alpha_{i}\frac{P_{uv,k}^{\left(i\right)}-{\tilde{\rho }}_{uv}^{\left(i\right)}}{n_{\lambda }^{\left(i\right)}}$$

where $P_{uv,k}^{\left(i\right)}$ is the difference of the measured distance between the navigation satellite with frequency k and satellite 1, 2. ${\tilde{\rho }}_{12}^{\left(i\right)}$ is the difference between the actual distance from satellite 1, 2, to the i-th satellite. λ is the reference frequency, $\alpha_{i}$ are the weights about each navigation satellite signal quality. As shown in the formula in Figure 10a, the phase error caused by the oscillator can be extracted from the carrier phase difference between GNSS receivers. This synchronization scheme requires accurate orbit determination data, and its effectiveness depends on whether the GNSS receiver will maintain the spectral purity inside the primary oscillator [74].

The GNSS-based phase synchronization scheme was further investigated in [40] through zero-baseline and short-baseline experiments. As shown in Figure 10b, the zero-baseline experiment refers to the GNSS signal received by a single antenna being distributed to two receivers via a splitter. The short-baseline experiment involves using two separate antennas to receive GNSS signals. Preliminary experimental results indicate that the GNSS-based phase synchronization method is feasible, but further experiments are needed for a comprehensive evaluation.

#### 3.3.2. MirrorSAR

**Figure 11:**
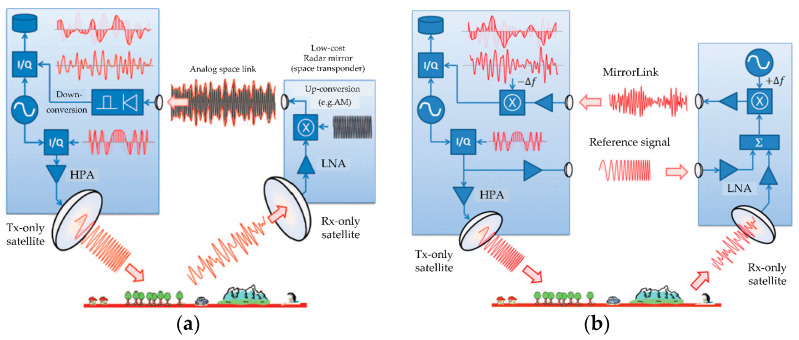
Potential synchronization links of Mirror SAR [18]: (**a**) a phase-preserving radar data link and (**b**) double-mirror synchronization.

The concept of MirrorSAR was proposed in [17,18,75]. The core of this concept is the greatly simplified receiver satellites, turning their main function into a kind of microwave mirror (space transponder), which returns the received radar echoes to one or more transmitters. The forwarded signals are then coherently demodulated at the transmitter using the same local oscillator. This design not only saves the hardware required for coherent demodulation, data storage, downlink, and digital control within the receiver satellite but also avoids the need for dedicated hardware links between transmitter and receiver to achieve phase synchronization, as with TanDEM-X and LuTan-1. For the MirrorSAR concept, two phase synchronization schemes are proposed. In the first simple configuration, the receiver’s function is merely to amplify and re-radiate the received echo signal. However, the transmitted signal may be interfered with by ground echoes. To solve this, a solution is proposed that both preserves the phase of the radar echo and avoids dependence on modulation carriers. As shown in Figure 11a, a high-frequency signal is generated by the receiver satellite, which is then modulated by the radar echo amplitude and transmitted to the transmitter satellite. Subsequently, amplitude demodulation occurs within the transmitting satellite to recover the echo signal from the high-frequency signal. The echo is demodulated by the transmitter’s oscillator, similar to a classical monostatic SAR [18].

In the second synchronization scheme, a double-mirror synchronization method is employed, as shown in Figure 11b. The transmitter satellite sends a reference signal to the receiver satellite. The receiver satellite simultaneously receives the echo signal and the reference signal. The overlaid signals are frequency shifted +∆f and re-radiated back to the transmitter satellite. The transmitter satellite performs a reverse frequency shift −∆f on the overlaid signals before demodulation. The phase error caused by modulated bistatic echoes is compensated by the modulation phase error (oscillator error) extracted from the reference signal during ground processing. The double-mirror synchronization method has been further studied in [76]. The reference defines the models of various error sources and gives the preliminary results of phase synchronization accuracy simulation. Reference [77] proposed an improved double-mirror synchronization scheme and evaluated the phase synchronization accuracy based on real TerraSAR-X data. Even with a synchronization signal-to-radar echo ratio as low as −7 dB, the proposed synchronization technique ensures a phase error estimation accuracy of less than 1°. A new constellation of geostationary and near-earth orbit SAR systems is proposed in [78], which achieves efficient revisit and phase/time synchronization for earth observation by using MirrorSAR technology.

Table 1 provides a summary of the phase synchronization methods analyzed during this chapter. It includes the application of the following synchronization methods and the feasibility of using the synchronization method in the multistatic SAR system.

## 4. Conclusions

The difference in oscillator frequency results in phase errors in the demodulated echo that vary along the azimuth time in the multistatic SAR systems. Therefore, phase synchronization is one of the important problems that has to be solved in multistatic systems. In this article, we reviewed the development of a multistatic SAR system. The basic principle of phase synchronization was introduced. The phase-synchronization methods were summarized. With the development of technology, future research will continue to explore more efficient and accurate phase synchronization methods to meet the growing demand for applications of multistatic SAR systems.

## Figures and Tables

**Table 1:** Summary of synchronization methods.

Synchronization Method		Application in Multistatic SAR	Feasibility
Direct microwave link	Continuous duplex	Not applied yet	The system hardware for phase synchronization can transmit and receive signals at the same time, and the signals must be sufficiently decoupled. The system design is complex.
	Pulsed duplex	Not applied yet	The method has the same system hardware and decoupling constraints as continuous duplex.
	Pulsed alternate	TandDEM-X, TianHui-2	The method has been applied in multistatic SAR. In order to transmit synchronization signals, bistatic SAR data acquisition is periodically interrupted, leading to periodic data loss.
	Noninterrupted pulsed alternate	LuTan-1	The method has been applied to the multistatic SAR. The method requires sufficient free time for exchanging synchronization pulses, so PRF design may cause problems in low frequency band multistatic SAR.
Data-based estimation algorithm	Automatic algorithm	TandDEM-X	The quality of the estimate depends on the quality and quantity of available measurements for residual phase errors in the image.
	Multisquint processing	TandDEM-X	
Evaluation of the raw data from the navigation receivers	GNSS-based phase synchronization	Not applied yet	This method requires accurate relative navigation data, and the GNSS receiver and radar payload share the same oscillator.
MirrorSAR	Phase-preserving radar data link	Not applied yet	Radar echoes may be affected by the reference synchronization signal, and the separation of the synchronization signal from the echo signal can be affected by the relative motion between satellites.
	Double mirror Synchronization	Not applied yet	

## Data Availability

No new data were created or analyzed in this study. Data sharing is not applicable to this article.
